# Survival Analysis of Cancer Patients of Differing Payer Type in South West Virginia, Between 2000 and 2013

**DOI:** 10.7759/cureus.3022

**Published:** 2018-07-22

**Authors:** Frank H Annie, Chris K Uejio, Abhishek Bhagat, Tanureet Kochar, Sarah Embrey, Alfred Tager

**Affiliations:** 1 Cardiology, Charleston Area Medical Center/Health Education and Research Institute, Charleston, USA; 2 Geography, Florida State Universiy, Charleston , USA; 3 Internal Medicne, Charleston Area Medical Center, Charleston, USA; 4 Internal Medicine, West Virginia University/Charleston Area Medical Center, Charleston, USA; 5 University of Charleston, School of Pharmacy, Charleston, USA; 6 Emergence Medicine, Charleston Area Medical Center, Charleston, USA

**Keywords:** payer, cancer, insurance

## Abstract

Introduction

The effect of insurance coverage on the health of at-risk populations is poorly understood in the Appalachian region of the United States. The goal of this study is to examine how different types of insurance coverage (Private Insurance, Medicare under 65, Medicare 65 or over, Medicaid and Self Pay) may influence cancer survival over time. This study analyzes colon, bladder, as well as combines anal, rectal, and esophageal cancers.

Methods

We systematically analyzed all West Virginia Cancer Registry patients between the years of 2000 and 2013 who was diagnosed with colon, bladder, anal, rectal, and esophageal cancers. Separate analysis examined colon (n = 927), bladder (n = 269), and combined anal, rectum, and esophageal cancers (n = 398). Cox proportional hazards models investigated the effect of insurance types on survival while controlling for age, sex, tobacco use, alcohol use, and cancer stage.

Results

Overall, tobacco use marginally significantly decreased colon cancer survival. Tobacco use had a suggestive relationship at hazards ratio at 1.150, 95% confidence interval: 0.9990-1.235, p = 0.052. The type of payer group did not alter survival. Older individuals tend to have a lower survival rate compared to those that are younger at the time of diagnosis. Also, late-stage cancer faced lower survival compared to those with early-stage cancer. Other results within stage groups corresponded to existing literature.

Conclusion

For the three differing cancer groupings, there was no significant survival difference for patients by insurance type. The effect of tobacco usage on colon cancer survival merits further research. The study design could be improved by considering more risk factors such as patient comorbidities that might affect patient care and survival.

## Introduction

The purpose of this study is to compare the survival of cancer patients stratified by type of insurance to determine if any significant disparities exist. Cancer treatment costs have increased dramatically over the last 30 years [1]. Being uninsured affects both the amount of time between diagnosis and treatment and course of treatment which can affect survival [2]. Chemotherapy can slow cancer progression and decrease the tumor mass by 1.5 to 2 cm; however, therapy is often expensive, creating barriers to care. Ensuring that patients are receiving equivalent care regardless of insurance category and the ability to pay is both an economic and ethical issue [3].

The type of health insurance can influence the type and duration of cancer treatments administered to patients. The list drug price is in the thousands of USD per year and would be prohibitively expensive for uninsured patients. Without comprehensive insurance, the drug treatment patients receive can dramatically affect their survival rate [4]. Unfortunately, data on the subject of insurance and survival outcomes for cancer chemotherapy patients is somewhat limited, especially in the study area in southern West Virginia [5]. The study examines colon, bladder, anal, esophageal, and rectal-based cancers. These soft tissue cancers were selected because they involve the digestive/gastrointestinal tract and are treated similarly.

Studies have examined disparities in cancer survival in patient subpopulations (race, ethnicity, age, and insurance types) [6-7]. Other studies focused on both broad cancer groups as well as narrow forms of cancer such as laryngeal cancer [8-9]. Relatively few studies focus on multiple cancer types that have similar mortality rates such as the present study [10]. Our study examines cancer survival in the understudied southern region of West Virginia. This study separately compares the median survival of colon, bladder, anal, rectal and esophageal cancer patients stratified by insurance category to determine if any disparities exist. The study controls for other independent survival risk factors such as the cancer stage at diagnosis, tobacco and alcohol use, gender, and age. We hypothesize that patients with public compared to private health insurance may have a greater colon, bladder, and anal-rectal cancer survival.

## Materials and methods

Study area, data, summary statistics

In this retrospective study, study subjects were identified using the Charleston Area Medical Cancer Center Registry and West Virginia Cancer Registry containing data between years 2000 and 2013. The geographical area covers the entire southern half of West Virginia as well as patients from the surrounding region (Ohio, Kentucky, and Virginia) who sought care at the non-profit Charleston Area Medical Center. Due to the large, non-profit structure, the hospital’s David Lee Cancer Center provides free/low-cost cancer care to uninsured patients that can prove the need for care. Thus, the study population primarily contains West Virginia patients but also includes people from other parts of Appalachia.

The study analyzed five groups of cancer with a corresponding international classification of disease codes listed in Table 1. Anal, rectal, and esophageal cancer cases were combined due to their low sample sizes and the similarities between these soft tissue cancers. Additionally, the treatment regimens for anal, rectal, and esophageal cancers are similar in protocol and drug usage. The cancer registry also provides patient demographic and risk factor information regarding sex, age, ever/never alcohol use, and ever/never tobacco use. The registry also recorded cancer stage at diagnosis and death, if surgery was performed or treatment refused, or when the patient left the study region. Patients were stratified into five health insurance groups: private insurance, Medicaid, Medicare age 65 or older, Medicare under the age of 65, and not insured/ self-pay. Age groups were divided into equal age segments that would best represent the data and the differing factors of risk of developing differing forms of cancer. Stage grouping was also considered. As this data was coded, it was then transferred to Stata 11.4 for analysis where a Cox proportional hazards model was created.

**Table 1:** ICD 9/10 Codes.

Cancer	ICD 9	ICD 10
Colon	153.9	C18.9
Bladder	188.9	C67.9
Anal	154.3	C21.0
Rectal	154.0	C20
Esophageal	150	C15.9

Patients with incomplete data relating to insurance type were removed from the study, including patients with the colon (N = 2267), bladder (N = 594), and anal, rectal and esophageal cancers (N = 1123). The study analyzed colon (N = 927), bladder (N = 284 cases), and combined anal, rectal, esophageal (N = 398) cancer survival. To understand if the demographic information was different from the patient group with incomplete data, the demographics of the original and reduced data were compared. After completing a summary of both sets of data, we found there is no major difference between these two groups. The data was evenly distributed among the three cancer. The primary outcome of median overall survival was assessed for each insurance category within each cancer type. Using a Cox proportional hazards models in Stata 11.4 compared differences in cancer survival after receiving a diagnosis while controlling for multiple independent risk factors and confounders. The Cox proportional hazards model assesses the hazard ratio of each group being tested as shown in Table 2.

**Table 2:** Demographic data and Cox proportional hazards outputs.

Category	Colon	Bladder	Anal, Rectal, and Esophagus
Male	441	189	249
Female	486	95	149
18–30	2	2	4
31–50	89	14	52
51–70	396	111	216
71–95	440	157	126
Private insurance	435	112	187
Medicaid	56	22	51
Medicare over the age of 65	378	134	125
Medicare under the age of 65	35	16	22
Not insured/Self Pay	23	0	13
Tobacco use	328	149	199
Alcohol use	862	259	63
Stage 0	8	27	26
Stage 1	155	179	99
Stage 2	269	32	93
Stage 3	272	23	105
Stage 4	223	23	75

## Results

**Figure 1:**
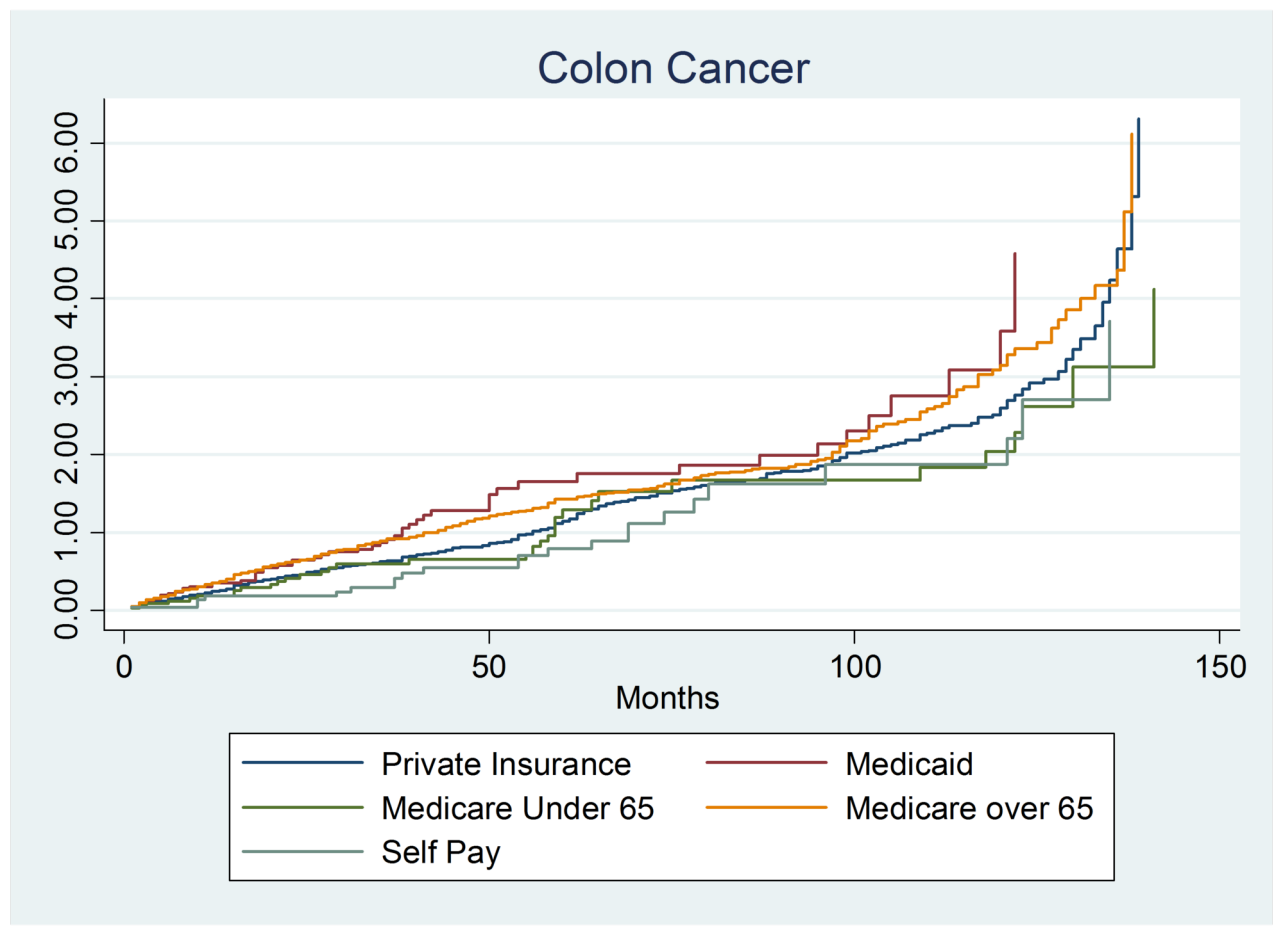
Colon cancer output.

We first examined whether colon cancer (n = 927) survival differed between payer groups, age, gender, and other risk factors. Overall, the type of insurance did not modify colon cancer survival after controlling for other risk factors. There were no significant differences in survival by gender or age groups (Figure 1). Of the other risk factors, there were only two statistically significant risk factors: tobacco usage and stage 4 disease. The tobacco usage hazards ratio was 1.150, 95% confidence interval of (.9990–1.235), and a p-value of 0.052. The other significant finding was stage 4 colon cancer with a hazards ratio of 4.075, the 95% confidence interval of (1.982–8.382) and a p-value of 0.001.

**Figure 2:**
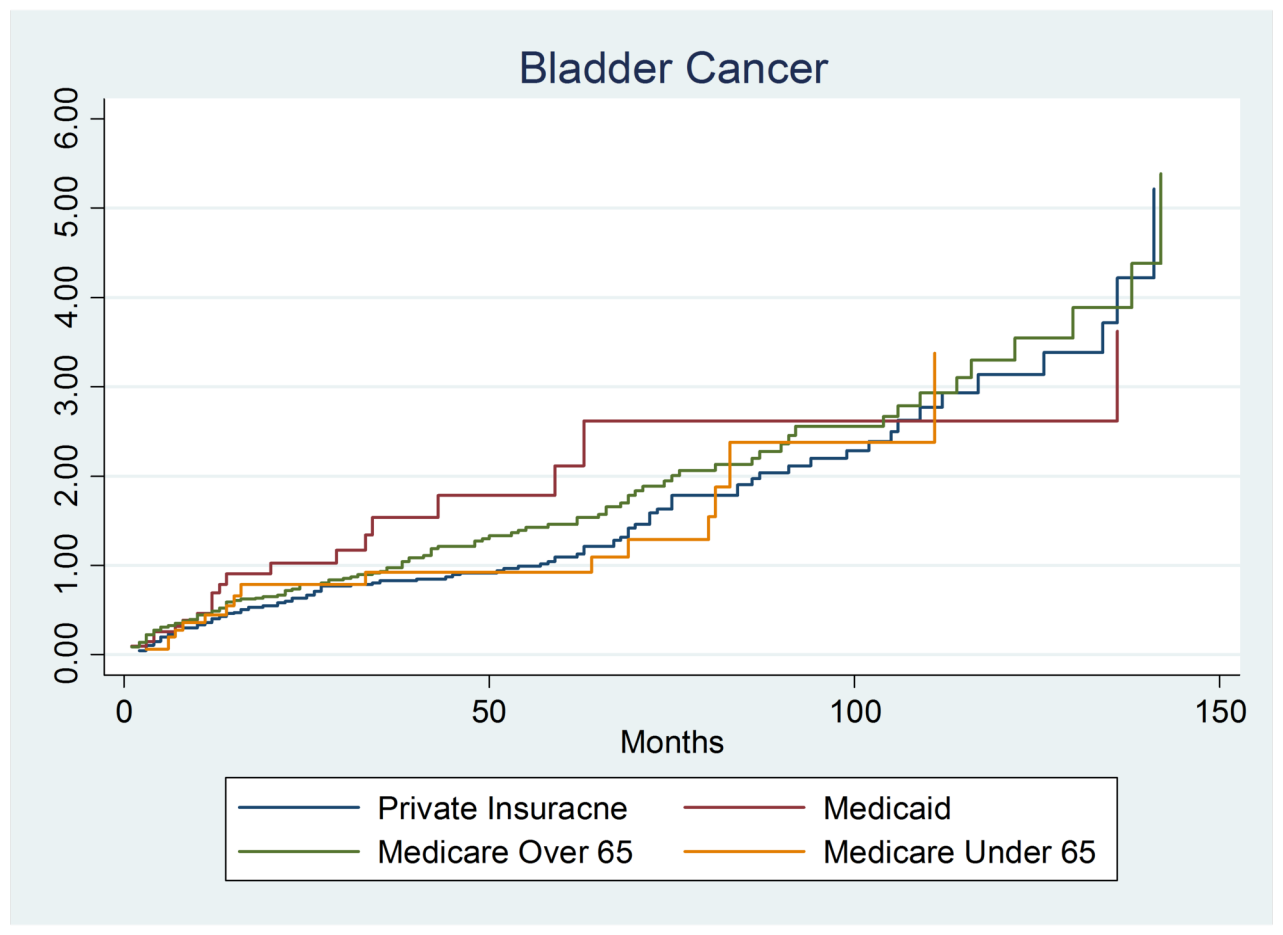
Bladder cancer output.

This section examined whether bladder cancer patients (n = 284) had differing survival rates. There was no evidence of difference in bladder cancer survival by payer group. Similarly, bladder cancer survival was not significantly influenced by patient demographics (gender or age). Similar to colon cancer, stage 2 and stage 4 bladder cancer had a significantly increased hazard (lower survival) than stage 0 patients. The stage 2 hazard ratio was 2.280, 95% confidence interval (1.294 – 4.017), and p-value = 0.004. The significant finding was stage 4 colon cancer with a hazards ratio of 3.418, 95% confidence interval of (1.891–6.178), and p-value of 0.001 as shown in Figure 2.

**Figure 3:**
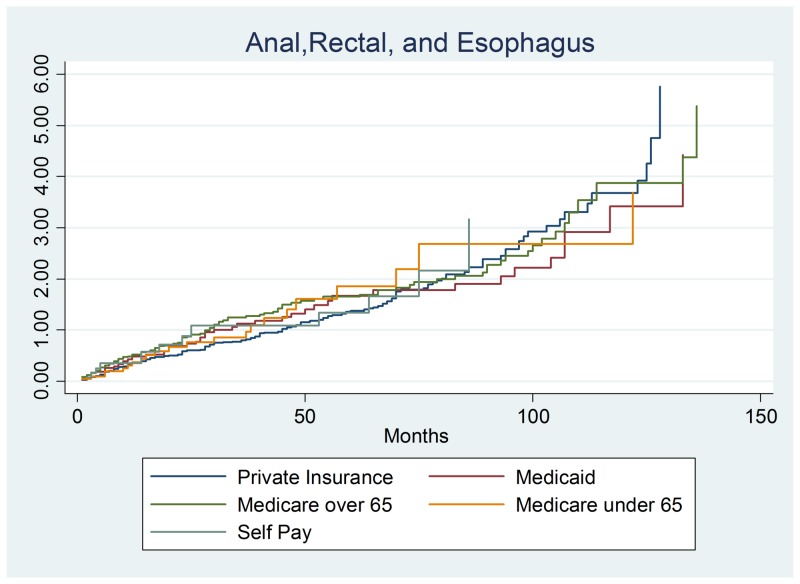
Anal, rectal, and esophageal cancer output.

The section reports the results of the combined anal, rectal and esophageal cancer (n = 398) survival analysis. These three cancers were combined in order to understand the overall impact of soft tissue cancers and to provide enough data points to perform this study. This section yielded similar results as the previous two sections as survival did not significantly differ by payer group. The only consistent risk factors were the cancer stage at diagnosis. Stage 1 had a protective hazards ratio of 0.0595, a 95% confidence interval of (0.3722–0.9021), and p-value of 0.016. Stage 2 cancer had a suggestive finding of a protective hazard of 0.6179, a 95% confidence interval of (0.3953–0.9658), and a p-value of 0.035. The final reportable finding was a statistically suggestive finding of stage 4 cancer with an increased hazard of 1.846, a 95% confidence interval of (1.166–2.923) and a p-value of 0.009. These results show that stage 1 and 2 diseases exhibited a lower hazard and a higher rate of survival than stage 0. Predictably, stage 4 cancer patients had increased hazards as the tumor grows rapidly and has spread to other organs (often liver and lungs) as shown in Figure 3 with total results listed in Table 3.

**Table 3:** Results of the three survival analyses. The table reports the hazard ratio, p-value, and 95% Confidence Interval for each independent variable.

Independent Variable	Colon	Bladder	Anal, Rectal, and Esophagus
Gender	0.9463 (0.420) (.8274–1.082)	1.223 (0.150) (.9300–1.609)	0.8142 (0.073) (.6504–1.019)
18–30	Comparison Group	Comparison Group	Comparison Group
31–50	1.650 (0.491) (.3968–6.868)	1.473 (0.617) (.3218–6.755)	1.021 (0.967) (.3654–2.858)
51–70	1.523 (0.561) (.3690–6.286)	1.663 (0.486) (.3977–6.960)	1.147 (0.788) (.4223–3.115)
71–95	2.178 (0.284) (.5249–9.038)	1.853 (0.401) (.4389–7.824)	1.984 (0.189) (.7136–5.518)
Private Insurance	Comparison Group	Comparison Group	Comparison Group
Medicaid	1.232 (0.155) (.9238–1.643)	1.375 (0.207) (.8383–2.256)	1.118 (0.498) (.8085–1.548)
Medicare under the age of 65	0.8131 (0.256) (.5689–1.162)	1.068 (0.674) (.7838–1.457)	0.8152 (0.152) (.6165–1.078)
Medicare over the age of 65	1.046 (0.563) (.8967–1.221)	1.098 (0.734) (.6372–1.894)	1.018 (0.937) (.6137–1.612)
Not insured/Self Pay	0.7996 (0.300) (.5237–1.220)	NA	1.348 (0.308) (.7590–2.396)
Tobacco Use	1.150 (0.052) (.9990–1.235)	1.109 (0.425) (.8591–1.433	1.140 (0.254) (.9098–1.429)
Alcohol Use	0.9933 (0.961) (.7615–1.295	0.6937 (0.104) (.4467–1.077)	0.7455 (0.048) (.5570–9978)
Stage 0	Comparison Group	Comparison Group	Comparison Group
Stage 1	1.148 (0.706) (.5591–2.359)	1.195 (0.401) (.7883–1.813)	0.5795 (0.016) (.3722–.9021)
Stage 2	1.383 (0.375) (.6753–2.832)	2.280 (0.004) (1.294–4.017)	0.6179 (0.035) (.3953–.9658)
Stage 3	1.525 (0.247) (.7465–3.115)	1.449 (0.212) (.8088–2.596)	0.9292 (0.744) (.5979–1.444)
Stage 4	4.075 (0.001) (1.982–8.382)	3.418 (0.001) (1.891–6.178)	1.846 (0.009) (1.166–2.923)

## Discussion

Overall results suggest that patients with colon, bladder, and anal, rectal, and esophageal cancers have similar mortality hazards across all payer groups. In the present study, tobacco decreased the survival of colon cancer patients. Tobacco use increases the risk of developing colon cancer. These results add further support to the existing literature [4, 11-14]. Consistent with the existing literature, stage 4 colon, bladder, anal, rectal, and esophageal cancer patients face higher hazards and lower survival compared to stage 0 patients. Advanced stage cancer patients may face additional complications (e.g., increased bleeding risk) or perhaps surgery-related complications that decrease survival [11, 13]. The standard treatment for bladder cancer is to remove the portion of the bladder at an earlier stage of diagnosis [11]. Stage 4 bladder cancers have several treatment options including chemotherapy, surgical treatment, and immunotherapy drugs. The final grouping was made up of anal, rectal, and esophageal cancers. Stage 2 is when cancer has spread through the muscle layer +/- nearby tissue. Stage 2B esophageal cancer can include spread to one or two nearby lymph nodes. Stage 4 is when cancer has spread to regional lymph nodes. The treatment would be to eradicate the muscle with the growth and the surrounding tissue. Stage 4 has completed spread to the original site and has moved to other organs within the body.

Other work, such as Giovannucci’s article, was an updated review of the epidemiological evidence of the links between cigarette smoking that might increase the risk of colorectal cancer [12]. Carcinogens from tobacco smoke enter the body from cigarettes and may damage the expression of several cancer-related genes. These genes could lead to increased mucous and an increase in adenomatous polyps within the colon. As Giovannucci’s work describes, 21 of 22 studies found that long-term heavy cigarette smokers have two to three times elevated risk of colon cancer development. Risk of cancer precursors was also elevated within smokers as well which illustrated further that smoking cigarettes increase all of the precursors of colon cancer in 12 of 12 studies provided in this study.

The present study found a supportive relationship between tobacco usage and an increased colon cancer hazards rate. Phipps et al. found smoking increases the risk of mortality by a hazards ratio of 1.30 compared to those without smoking history of a hazards ratio of 0.96 [15]. Other work includes Pelser et al.’s study which recorded a higher relative risk of 1.46 for those who did smoke associated with colon cancer [16]. Additional works such as of Zhu et al. also illustrated that those who smoke cigarettes have a higher hazards ratio of 1.72 within the papers study population [17]. To further explore the impact of the connection between smoking and colon cancer, Nordenvall (2013) study was also reviewed which included a sizeable 40,230-person cohort study on Swedish population and the role of tobacco products on incidents of cancer within that population. Overall, the existing literature expresses that within multiple study populations, those with cigarette use have an increased relative risk of colon cancer as well as the lower survival of the overall population associated with cigarette smoking over a long period. Slattery et al. observed a 50% increase in colon cancer risk within the population that smoked cigarettes [18-20]. This study also illustrated that those with heavier usage would have a greater risk of smoking more than one pack, or 20 cigarettes per day and a larger body mass as well.

## Conclusions

Overall, patients with different insurance types have a similar survival rate over time. Insurance types can influence the amount and quality of health care and patient survival. The study examined three different types of cancer (lung, breast, and prostate cancer) and different payer types. Cancer incidence rates within Appalachia are statistically higher than other regions between the years of 1980 and 2014. Our results can provide survival prognosis information for newly diagnosed cancer patients. This study also provides baseline cancer hazard risks from years 2000 to 2013 for future studies of novel cancer treatments. We were unable to examine a more extended study period since the cancer registry did not systematically collect health insurance information until the year 2000. The study did not look at racial or ethnic disparities since this region is 97% white non-Hispanic. The research could have been improved by examining potential socioeconomic disparities, but the cancer registry did not routinely collect this information.
